# Tackling aggression: Translating preclinical insights into clinical relevance

**DOI:** 10.1002/ctm2.70334

**Published:** 2025-05-02

**Authors:** Mingyue Lv, Scott J. Russo, Long Li

**Affiliations:** ^1^ State Key Laboratory of Cognitive Science and Mental Health, Institute of Biophysics, Chinese Academy of Sciences Beijing China; ^2^ University of the Chinese Academy of Sciences Beijing China; ^3^ Nash Family Department of Neuroscience, Friedman Brain Institute, Icahn School of Medicine at Mount Sinai New York New York USA; ^4^ Brain and Body Research Center, Icahn School of Medicine at Mount Sinai New York New York USA

## 1 AGGRESSION AS A CLINICAL CHALLENGE ACROSS BRAIN DISORDERS

Aggression is a clinically significant and often disruptive behavioural symptom that spans a wide range of brain disorders. Despite distinct pathologies, both psychiatric and neurodegenerative diseases frequently manifest aggression, complicating disease management, threatening safety and worsening long‐term outcomes.^1^

In psychiatric disorders, particularly schizophrenia, aggression is frequently observed during acute psychotic episodes marked by paranoia, hostility, and agitation. In inpatient settings, the prevalence of aggression among individuals with schizophrenia ranges from 10% to over 50%.^2^ Broader epidemiological studies, such as the Epidemiologic Catchment Area project, have demonstrated that individuals with schizophrenia, bipolar disorder, mania or major depression are approximately five times more likely to engage in violent acts—a figure that increases 16‐fold when substance abuse is also present.^3^

Aggression is not limited to psychiatric disorders. It is also a prevalent and challenging symptom in neurodegenerative conditions such as Alzheimer's disease (AD). Nearly 28% of patients with AD and around 7% of those with mild cognitive impairment exhibit aggressive behaviours, including verbal hostility, physical aggression and resistance to care.^4^ Aggression is a leading cause of institutionalization, with one study reporting that 34.2% of dementia‐related hospitalizations were triggered by aggressive episodes.^5^ Notably, male patients tend to display higher levels of physical and verbal aggression, alongside other disruptive behaviours such as disinhibition and wandering.^6^

Taken together, these findings underscore aggression as a transdiagnostic symptom that cuts across disease boundaries. Its prevalence across disparate conditions suggests the involvement of shared neural substrates, warranting further investigation into the underlying circuit and molecular mechanisms. Such insights could pave the way for novel interventions that target aggression regardless of disease category.

## 2 A CRUCIAL ROLE FOR THE CORTICAL AMYGDALA IN SHAPING AGGRESSION

Our recent study, published in *Nature*,^7^ uncovers a previously unrecognized neural circuit centered on estrogen receptor 1‐expressing (Esr1⁺) neurons within the posterolateral cortical amygdala (COApl) that selectively regulates male aggression. Using an integrative approach—encompassing whole‐brain cFos mapping, in vivo calcium imaging, viral tracing and genetic perturbation—we found that Esr1^COApl^ neurons act as a key node encoding the motivational drive for aggression.

Activity mapping revealed that the COApl is consistently activated in aggressive male mice, acting as a functional hub that orchestrates coordinated neural activity across multiple aggression‐related regions. In‐vivo fiber photometry further demonstrated that Esr1^COApl^ neurons are not only active during the act of aggression but also during the anticipatory phase of a social investigation that precedes the attack. This temporal pattern suggests these neurons encode the motivational salience or perceived threat level of social stimuli, rather than aggression per se.

**FIGURE 1:**
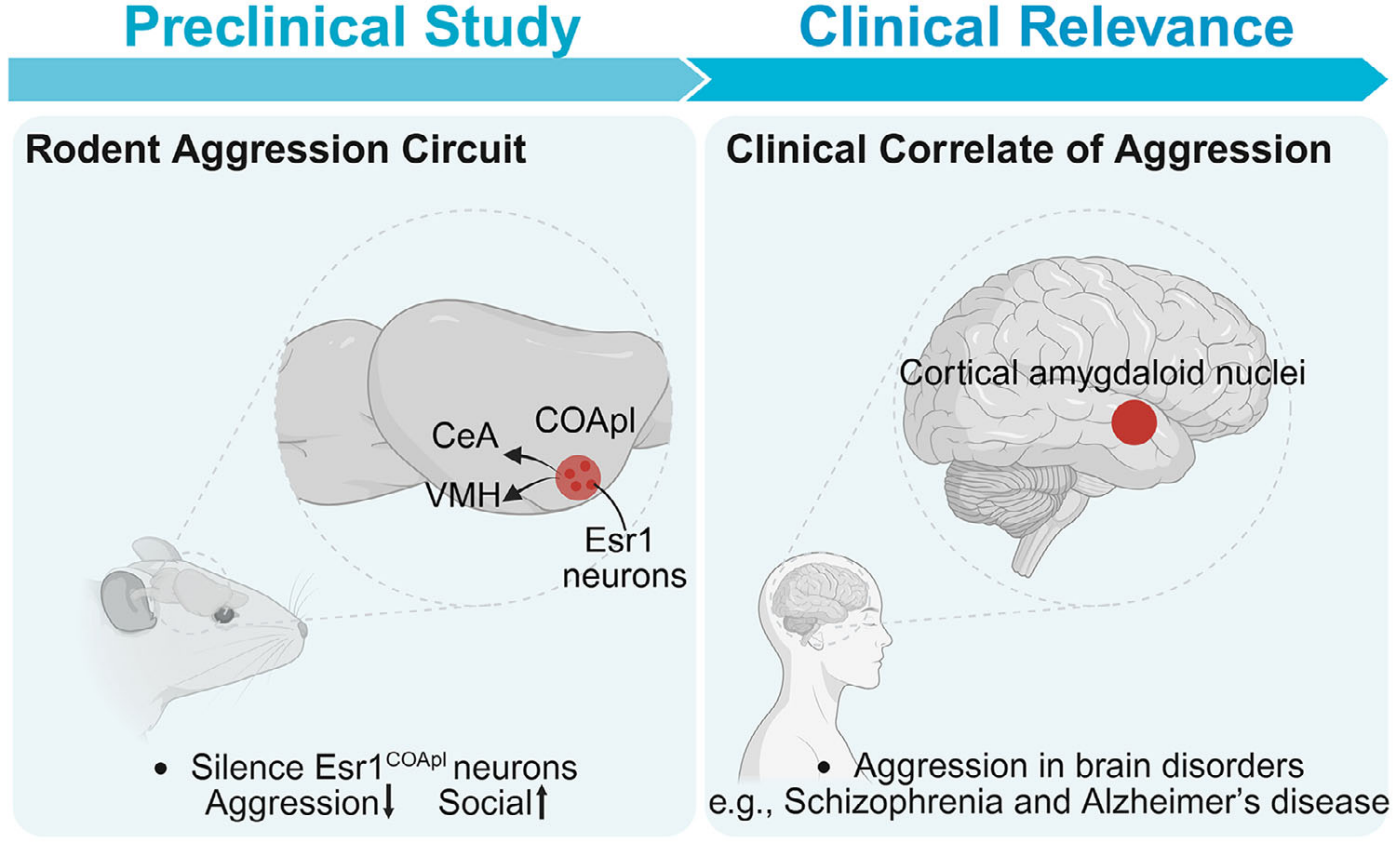
Schematic illustration of aggression‐regulating circuits in mice and their potential clinical relevance in humans (created with BioRender.com). (Left) In mice, estrogen receptor 1‐expressing (Esr1⁺) neurons in the posterolateral cortical amygdala (COApl) regulate aggression via projections to the ventromedial hypothalamus (VMH) and central amygdala (CeA), suppressing aggression while enhancing social behaviours. (Right) Inspired by these findings, the human cortical amygdaloid nuclei—may represent a target for future translational studies on aggression in psychiatric and neurodegenerative disorders.

Importantly, chemogenetics and optogenetics silencing of Esr1^COApl^ neurons led to a robust and selective suppression of aggression, while simultaneously enhancing prosocial behaviours such as close investigation and affiliative sniffing. Crucially, these manipulations did not impair social reward and reinforcement behaviour, indicating that Esr1^COApl^ neurons play a specific role in aggressive motivation without broadly disrupting social function. Anatomically, we found that Esr1^COApl^ neurons project to two key downstream targets ventromedial hypothalamus and central amygdala. Both projections were functionally necessary for the aggression‐promoting effects of the COApl, supporting the idea that this circuit exerts top‐down control over deeper subcortical aggression centers (Figure 1).

## 3 CLINICAL IMPLICATIONS AND FUTURE DIRECTIONS

Clinically, one of the most pressing challenges is how to mitigate pathological aggression without impairing normal social functioning. The discovery of an Esr1^COApl^‐centered circuit that specifically governs aggressive motivation—but spares prosocial behaviour—offers a promising solution. Unlike conventional pharmacological interventions such as antipsychotics or selective serotonin reuptake inhibitors, which often blunt social engagement, targeted modulation of this pathway reduced aggression while preserving, and even enhancing, social interaction. This circuit‐level dissociation of aggression from sociability also sheds light on sex‐specific mechanisms of aggression. The Esr1^COApl^ pathway was found to be selectively active in male mice, implicating sex hormones and dimorphic neural architecture in shaping how the brain encodes social threats. These findings may help explain the marked male predominance observed in aggression‐related disorders such as antisocial personality disorder and further suggest that sex‐tailored interventions could be both necessary and feasible in clinical practice.

From a translational perspective, the COApl is part of the conserved “ cortical amygdaloid nuclei ” in humans, which is implicated in the generation of learned and innate behaviours, as well as positive and negative valence processing.^8^ This cross‐species conservation enhances the translational relevance of our findings and supports the feasibility of non‐invasive imaging of homologous human circuits. Advances in high‐resolution functional magnetic resonance imaging and positron emission tomography imaging may enable the development of circuit‐based biomarkers to detect aggression‐related dysfunction, stratify patients, predict treatment responses or inform personalized care plans. In parallel, the involvement of ESR1 introduces a neuroendocrine dimension to aggression regulation. Meta‐analytic data suggests a positive association between estradiol levels and aggression in humans,^9^ and our data provide a cellular substrate for this relationship. These findings open the door to exploring hormonal therapies or selective ESR1 modulators as precision treatments for individuals with endocrine‐related aggression phenotypes or hormone‐sensitive conditions.

Looking forward, it will be important to determine whether analogous or distinct circuits govern aggression in females. Although less frequently studied, female aggression is clinically relevant—particularly in contexts such as trauma, mood disorders or postpartum psychiatric syndromes. Bridging our preclinical findings to human studies—through neuroimaging, behavioural phenotyping, and longitudinal approaches—will be essential for clinical translation.

## References

[1] Girasek H , Nagy VA , Fekete S , Ungvari GS , Gazdag G . Prevalence and correlates of aggressive behavior in psychiatric inpatient populations. World J Psychiatry. 2022;12(1):1‐23. doi:10.5498/wjp.v12.i1.1 35111577 PMC8783168

[2] Cornaggia CM , Beghi M , Pavone F , Barale F . Aggression in psychiatry wards: a systematic review. Psychiatry Res. 2011;189(1):10‐20. doi:10.1016/j.psychres.2010.12.024 21236497

[3] Swanson JW , Holzer CE, 3rd , Ganju VK , Jono RT . Violence and psychiatric disorder in the community: evidence from the Epidemiologic Catchment Area surveys. Hosp Community Psychiatry. 1990;41(7):761‐770. doi:10.1176/ps.41.7.761 2142118

[4] Yu R , Topiwala A , Jacoby R , Fazel S . Aggressive behaviors in Alzheimer disease and mild cognitive impairment: systematic review and meta‐analysis. Am J Geriatr Psychiatry. 2019;27(3):290‐300. doi:10.1016/j.jagp.2018.10.008 30527275 PMC6399100

[5] Takacs R , Ungvari GS , Gazdag G . Reasons for acute psychiatric admission of patients with dementia. Neuropsychopharmacol Hung. 2015;17(3):141‐145. 26485744

[6] Ott BR , Lapane KL , Gambassi G . Gender differences in the treatment of behavior problems in Alzheimer's disease. SAGE study group. Systemic assessment of geriatric drug use via epidemiology. Neurology. 2000;54(2):427‐432. doi:10.1212/wnl.54.2.427 10668707

[7] Aubry AV , Durand‐de Cuttoli R , Karpman E , et al. A crucial role for the cortical amygdala in shaping social encounters. Nature. 2025;639(8056):1006‐1015. doi:10.1038/s41586-024-08540-4 39939764 PMC11946885

[8] Sosulski DL , Bloom ML , Cutforth T , Axel R , Datta SR . Distinct representations of olfactory information in different cortical centres. Nature. 2011;472(7342):213‐216. doi:10.1038/nature09868 21451525 PMC3354569

[9] Wang Y , Wang H , Cai J , et al. Association between estradiol and human aggression: a systematic review and meta‐analysis. Psychosom Med. 2023;85(9):754‐762. doi:10.1097/PSY.0000000000001247 37678333 PMC10662589

